# Integrating machine learning to advance epitope mapping

**DOI:** 10.3389/fimmu.2024.1463931

**Published:** 2024-09-30

**Authors:** Simranjit Grewal, Nidhi Hegde, Stephanie K. Yanow

**Affiliations:** ^1^ Department of Medical Microbiology and Immunology, University of Alberta, Edmonton, AB, Canada; ^2^ Department of Computing Science, University of Alberta, Edmonton, AB, Canada; ^3^ School of Public Health, University of Alberta, Edmonton, AB, Canada

**Keywords:** machine learning, epitope, B-cell, algorithm, features, databases, toolboxes, vaccine

## Abstract

Identifying epitopes, or the segments of a protein that bind to antibodies, is critical for the development of a variety of immunotherapeutics and diagnostics. In vaccine design, the intent is to identify the minimal epitope of an antigen that can elicit an immune response and avoid off-target effects. For prognostics and diagnostics, the epitope-antibody interaction is exploited to measure antigens associated with disease outcomes. Experimental methods such as X-ray crystallography, cryo-electron microscopy, and peptide arrays are used widely to map epitopes but vary in accuracy, throughput, cost, and feasibility. By comparing machine learning epitope mapping tools, we discuss the importance of data selection, feature design, and algorithm choice in determining the specificity and prediction accuracy of an algorithm. This review discusses limitations of current methods and the potential for machine learning to deepen interpretation and increase feasibility of these methods. We also propose how machine learning can be employed to refine epitope prediction to address the apparent promiscuity of polyreactive antibodies and the challenge of defining conformational epitopes. We highlight the impact of machine learning on our current understanding of epitopes and its potential to guide the design of therapeutic interventions with more predictable outcomes.

## 1 Introduction

Vaccines are among the most successful and cost-effective public health interventions, particularly to protect against infectious diseases. This was never more evident than during the COVID-19 pandemic where vaccines were the most valuable intervention to protect vulnerable populations from hospitalization and death (1, 2). All the SARS-CoV-2 vaccines were based on the spike protein as the vaccine antigen, either expressed from DNA, mRNA, or as a recombinant protein, and elicited immune responses against dominant epitopes (or segments) within the protein. In response, new variants of the virus emerged with different amino acid sequences in these epitopes, impairing the efficacy of the first-generation vaccines and requiring the design of new variant-specific ones. The ongoing management of SARS-CoV-2 depends on our preparedness against emerging variants; this can be facilitated by designing vaccines that focus immune responses on highly conserved epitopes.

Precise mapping of B-cell epitopes is critical beyond vaccine development for other antibody-based interventions such as immunotherapeutics and diagnostics (3, 4). Likewise, mapping T-cell epitopes can improve our understanding of immune responses to infectious and autoimmune diseases (5) and support the development of immunotherapeutics with an increased safety profile by reducing off-target effects (6). The challenge is how to identify or map these epitopes.

Machine learning algorithms targeted at epitope mapping are undergoing continual development and immense growth. These algorithms are improving upon existing *in vitro* methods by exploiting the vast reservoir of published experimental data to find patterns and predict regions of a protein likely to be a part of an epitope. Here, we discuss how current methods have benefited from integrating machine learning and explore future applications to further refine epitope mapping.

## 2 *In vitro* epitope mapping methods

What constitutes an epitope varies immensely between two major types of immune cells in the body: T-cells and B-cells. T-cell epitopes consist of antigens processed into 8-10 amino acid linear segments that are recognized by major histocompatibility complex (MHC) class I molecules and 13-17 amino acid segments for MHC class II molecules (7). In contrast, B-cell epitopes are typically (90%) conformational, involving amino acids that are spatially close to one another due to secondary structure, tertiary structure, or quaternary structure (8–10). This makes them variable in length and structure. Further, while antibodies typically interact with 15 to 22 amino acids on the surface of an antigen, approximately 5 amino acids contribute most significantly to stabilizing the antibody-antigen complex (11).

Several experimental methods are used to map epitopes, each with pros and cons related to accuracy, throughput, and cost (12). X-ray crystallography, for example, provides accurate information about the three-dimensional complex between an antibody and the antigen that can encompass conformational changes and even highlight dynamics by comparing bound and unbound states. Epitopes are mapped based on their proximity to crystallized residues within the paratope of the antibody (13). Analysis tools, such as PDBePISA, provide information on the interactions of residues between an epitope and the corresponding paratope based on distance, participating residues, and orientation (14). Challenges with this approach are the stability, size, and solubility of the proteins to form a well-ordered lattice. Post-translational modifications can also impede crystal formation. Further, there can be several possible antibody-antigen complexes depending on the physical parameters used for crystallization (15).

Cryo-electron microscopy (cryo-EM) is another similar biophysical method for mapping epitopes. Freezing epitopes bound and unbound to their paratope yields density maps that compare the two states. These density maps are made by compositing 2D images from various angles into a 3D map of the molecule and the differences between the two density maps indicate the residues of an epitope (16).

On the other hand, screening peptide arrays provides much higher throughput, but at the cost of accuracy; the resolution of an epitope by a peptide array is not as high as the resolution attained from cryo-EM or X-ray crystallography. Peptide arrays consist of libraries of synthetic, overlapping peptides (usually 15-20 amino acids in length) that are screened with an antibody of interest (or serum) to identify those peptides that bind strongly (17). Thousands of peptides can be screened at once. The primary antibody bound to peptides is detected by a secondary antibody that emits a luminescent signal. Despite epitopes being typically conformational, peptide arrays are useful for several reasons (9). They are ideal for mapping linear epitopes and the extent of overlap among the peptides can reveal residues that are important components of the epitope. These can be specifically mutated (e.g. by alanine walking) to confirm their contribution to antibody binding (18). Thus, it is possible to determine key residues that dominate the antibody’s affinity for the linear epitope (19). However, peptide arrays are less informative to identify conformational epitopes. When screening reveals multiple peptides recognized by an antibody, the interpretation of these data relies on 3D models of the protein to map the binding sites. If the reactive peptides lie in proximity within the 3D protein structure, this could indicate a conformational epitope (20).

## 3 Machine learning

Machine learning is a powerful tool that can be used to address the complexity of data from epitope mapping studies. It is effectively a subset of artificial intelligence in which previous outcomes of a task provide an algorithm with experience that allows it to improve in the same task applied to new data (21). ‘Surviving the Titanic’ is a common example of machine learning; this problem involves a dataset containing passenger information that is used to predict the likelihood of survival of specific passengers (22). Information on gender, age, cabin class, etc. are studied and used to predict whether a passenger survived the Titanic disaster. The algorithm identifies patterns in the data and correlates them to what it is tasked with predicting (in this case survival). The ‘Surviving the Titanic’ problem is a valuable practice problem to learn the basics of machine learning and includes many resources and tutorials.

### 3.1 Datasets – training and testing

Importantly, the performance of an algorithm directly relates to the breadth and complexity of the datasets it learns from. The more novel cases and rare ‘edge’ cases in the dataset, the more the algorithm will learn how to predict similar scenarios in the future. In this way, machine learning can be used to make accurate predictions on a variety of tasks. In general, the algorithm benefits from more data but it is also important to recognize that curated data may be valuable for developing specialized algorithms. For example, if a machine learning algorithm is used specifically to predict cancer epitopes, it should be trained on a specialized cancer database, such as CEDAR (**Table 1**) (23). Adding data for the prediction of different types of epitopes may improve a machine learning algorithm’s generalizability but could also mask biological patterns unique to cancer epitopes. Additionally, data that are very similar to existing training data do not ‘teach’ an algorithm anything new.

**Table 1:** T-cell epitope associated databases^a^.

#	Name	Scope	Approximate size	Link	Ref.
1	IEDB-3D 2.0	Structural T-cell & B-cell epitopes	410 assays (T-cell) 1,446 assays (MHC binding)	https://www.iedb.org/	(81)
2	HIV molecular immunology database	HIV-associated T-cell & B-cell epitopes	13,700 entries (T-cell)	https://www.hiv.lanl.gov/content/immunology/index.html	(82)
3	CEDAR	Cancer-associated T-cell & B-cell epitopes	224,000 entries (B-cell & T-cell)	https://cedar.iedb.org/	(23)
4	IEDB	T- cell & B-cell epitopes	1,610,000 entries (B-cell & T-cell)	https://www.iedb.org/	(83)
5	TANTIGEN 2.0	Cancer-associated T-cell epitopes	1,000 entries	http://projects.met-hilab.org/tadb/	(84)
6	Protegen	Protective antigens	1600 entries	https://violinet.org/protegen/	(85)
7	FLAVIdB^b^	Flavivirus associated T-cell & B-cell epitopes	12,800 entries (B-cell & T-cell)	http://cvc.dfci.harvard.edu/flavi/	(86)
8	MHCBN 4.0	Peptides associated with TAP and MHC	25,000 entries	https://webs.iiitd.edu.in/raghava/mhcbn/	(87)
9	EPIMHC	MHC-restricted peptide ligands and epitopes	4800 entries	http://bio.med.ucm.es/epimhc/	(88)
10	SYFPEITHI	Peptide binding MHC I or MHC II	7,000 entries	http://www.syfpeithi.de/	(89)

^a^Accessibility evaluated on 12-06-2024.

^b^This database was found to be inaccessible as of 12-06-2024. Its inclusion depends on two factors: links often migrate or are repaired, and these databases have been used in the development of tools in the past. The link provided is the last known link used to access the database.

Many publicly accessible databases are available as sources of datasets for either T-cell or B-cell epitopes (**Tables 1**, **2**). These databases provide large, specialized sources of data that algorithms can study and use for predictions. RCSB PDB, for example, provides primarily 3D structures of various proteins and multimolecular complexes, and information relating to their molecular composition, position, length, chains, etc. AntigenDB, on the other hand, provides sequence, structure, classifications, etc., of various experimentally validated antigens. AntiJen contains data on many topics but, notably, it is a valuable source of data for both B-cell and T-cell epitopes.

**Table 2:** B-cell epitope associated databases^a^.

#	Name	Scope	Approximate size	Link	Ref.
1	IEDB-3D 2.0	Structural T-cell & B-cell epitopes	4,859 assays (B-cell)	https://www.iedb.org/	(81)
2	SDAP 2.0	Allergen-associated B-cell epitopes	4,000 entries	https://fermi.utmb.edu/SDAP/sdap_fas.html	(90)
3	HIV molecular immunology database	HIV-associated T-cell & B-cell epitopes	4,200 entries (B-cells)	https://www.hiv.lanl.gov/content/immunology/index.html	(82)
4	CEDAR	Cancer-associated T-cell & B-cell epitopes	224,000 entries (B-cell & T-cell)	https://cedar.iedb.org/	(23)
5	IEDB	T-cell & B-cell epitopes	1,610,000 entries (B-cell & T-cell)	https://www.iedb.org/	(83)
6	FLAVIdB^b^	Flavivirus-associated T-cell & B-cell epitopes	12,858 entries (B-cell & T-cell)	http://cvc.dfci.harvard.edu/flavi/	(86)
7	Protegen	Protective antigens	1600 entries	https://violinet.org/protegen/	(85)
8	CED^b^	Conformational B-cell epitopes	225 entries	http://immunet.cn/ced/	(91)
9	EPITOME^b^	B-cell epitopes	142 entries	https://www.rostlab.org/services/epitome/submit.php	(92)
10	BciPep	B-cell epitopes	3,031 entries	http://crdd.osdd.net/raghava/bcipep/pep_src.html	(28)
11	AntiJen	T-cell & B-cell epitopes	24,000 entries (B-cell & T-cell)	http://www.ddg-pharmfac.net/antijen/AntiJen/antijenhomepage.htm	(93)

^a^Accessibility evaluated on 12-06-2024.

^b^This database was found to be inaccessible as of 12-06-2024. The choice to include it depended on two factors: links often migrate or get repaired and these databases have been used in the development of tools in the past. The link provided is the last known link used to access the database.

In essence, a machine learning algorithm studies databases to find patterns that inform the analysis of novel data. The quality of the algorithm requires careful selection of the appropriate database. For example, EPSVR is a B-cell epitope prediction algorithm focused on conformational epitopes and the type of data needed for accurate conformational epitope prediction is structural (24). Their training set consisted of structures of curated antigen-antibody complexes and was tested on a sampling of structures from the Conformation Epitope Database (CED) (25, 26). By limiting their datasets to a particular type of epitope, the algorithm becomes much more accurate at predicting that type of epitope. In the case of EPSVR, linear epitopes were excluded because of their poor correlation to structural data. If linear epitopes were included, they may affect how the algorithm evaluates structural data and decrease prediction accuracy on conformational epitopes. For LBTOPE, a model focused on linear epitope prediction, only the primary sequences of epitopes for B-cell receptors and non B-cell epitopes are needed (27). Since these data are more available and less complex, it is easier to generate a dataset of tens of thousands of entries. In another example, the BCIpep database contains many linear epitopes and was used to develop ABCpred to support peptide-based vaccine design and allergy research (28, 29). Training the dataset specifically on linear epitopes resulted in a model that can strongly predict these types of epitopes.

Finally, datasets are further divided into ‘training sets’, ‘validation sets’, and ‘test sets’. Machine learning models are built from training sets; a validation sets help determine the optimal parameters or models for a given problem; and test sets are used to evaluate the machine learning models. These sets are mutually exclusive.

### 3.2 Features and labels – how data are encoded

Even when an algorithm is provided a database robust enough to characterize the different patterns present in both linear and conformational epitopes, it still requires the tools to make use of those data. These databases are assembled into datasets with each datapoint referred to by the term ‘example’. Within each example there is information relating to ‘labels’ and ‘features’. Labels and features are the tools an algorithm uses to analyze examples.

Labels are what the algorithm learns to predict (the outcome). For instance, the label may refer to the strength of peptide-antibody binding. Machine learning can predict labels based on classification or regression analysis (30); for B-cell epitope prediction, classification involves classifying a peptide as a B-cell epitope or not, while regression analysis would assign a continuous value to the likelihood of the binding.

**Figure 1:**
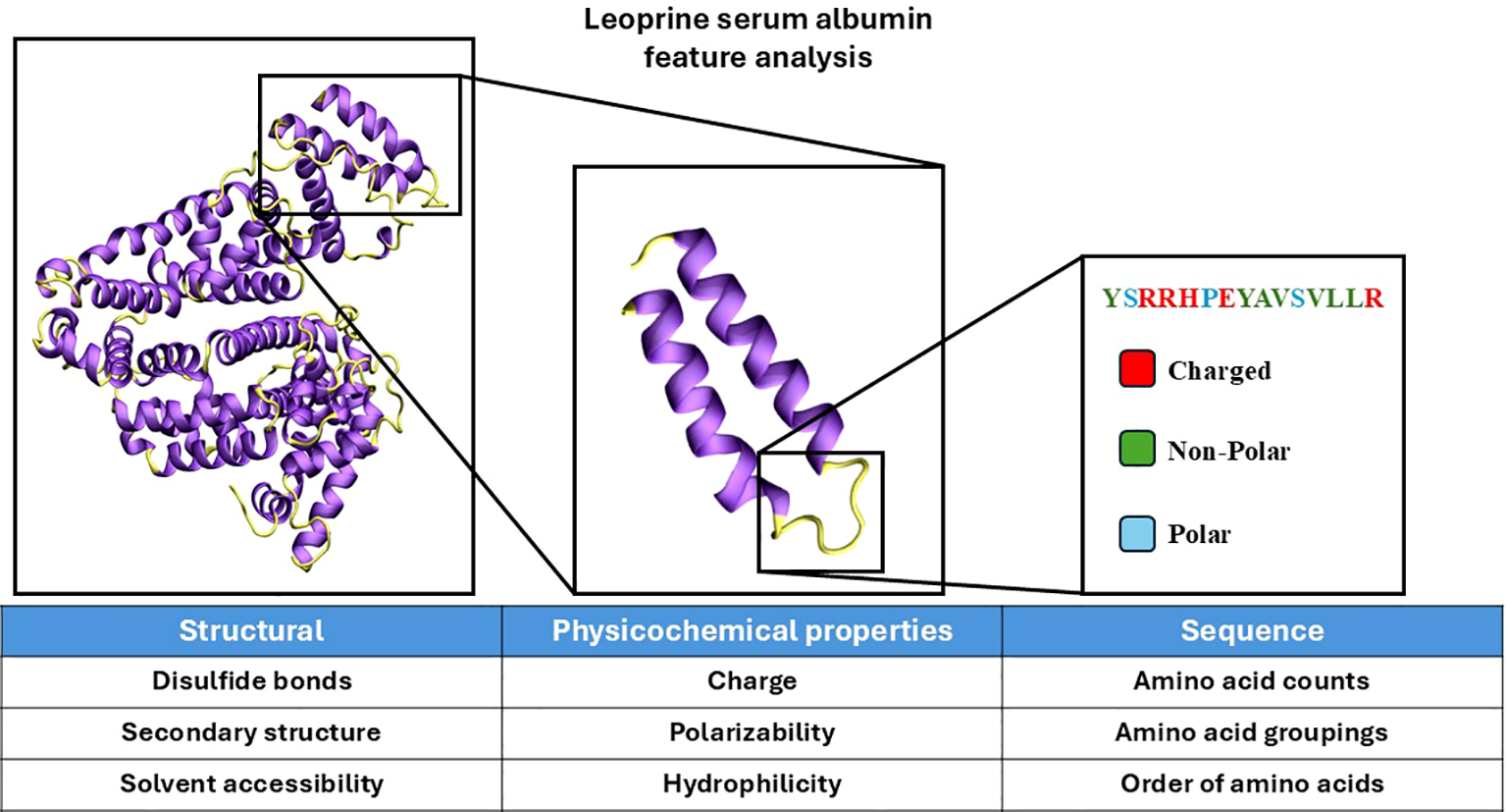
Visualization of protein analysis for feature design. The protein structure of leporine serum albumin was visualized in ‘new cartoon’ with VMD (33). Coil and loop regions of the secondary structure are colored yellow and helical regions are colored purple. Certain features are derived from the analysis of the whole protein, smaller subsections, and sequence-based analysis. Features can broadly be categorized as structural, physicochemical, or sequence-based. The features provided below each of the categories are select examples that belong to each category. The secondary structure used as referenced PDB ID 4F5V (34).

Features contain descriptive information about the peptide (sequence, structure, physico-chemical properties, etc.) or the parent protein it is derived from. The learning algorithm effectively learns patterns in the features that relate to a specific label value. More specifically, several algorithms use surface accessibility as the feature to predict whether an epitope is likely to be recognized by B-cells as this feature correlates well with binding strength (the label) (31, 32). Developing accurate machine learning models begins with selecting features that correlate strongly to a label (30). The features that are important depend entirely on the nature of the question being asked and the success of an algorithm depends on the design of features (**Figure 1**). A good feature set uses all data that correlate well with the label (what is being predicted). However, there are many ways to encode the same feature that are more effective for specific machine learning algorithms (**Table 3**).

**Table 3:** Features employed by machine learning-based B-cell epitope prediction tools.

Machine learning tool	Feature types	Year	Ref.
ABCpred	Amino acid composition and sequence	2006	(29)
COBEPRO	Similarity to other epitopes	2009	(94)
EPITOPIA	Amino acid preference, secondary structure preference, surface accessibility, surface structure, evolution rate, polarity scale, flexibility scale, antigenicity scale, hydrophilicity scale	2009	(37)
CBTOPE	Amino acid composition, polarity, flexibility, antigenicity, hydrophobicity, sequence, similarity to other epitopes	2010	(36)
LBtope	Amino acid composition, sequence, similarity to other epitopes, variable epitope length control	2013	(27)
SEPPA 1.0 (*) SEPPA 2.0 (**) SEPPA 3.0 (***)	Amino acid propensity*, sequence combined with structure*, solvent accessible surface areas*, antigenicity combined with structure **, glycosylation combined with structure***	2019	(95–97)
EPCES/EPSVR	Amino acid, side-chain energy score, surface exposure, antigenicity combined with surface structure, and secondary structure	2020	(24)
SCANNET	Amino acid composition, secondary structure, accessible surface area, coordination number (van der Waals interaction), 2D solvent exposure, backbone and sidechain depth (distance from surface), surface convexity index, amino acid conservation	2022	(32)

Different versions of the SEPPA program have new features added to them, features with '*' were first included in SEPPA 1.0, while features with '**' and '***' were first included in SEPPA 2.0 and SEPPA3.0 respectively.

For example, algorithms EPITOPIA and CBTOPE both use Grantham polarity and Ponnuswami polarity index to calculate polarity as a feature, while LBtope cites CBTOPE’s feature set but excludes polarity entirely (35–37). This may be because LBtope is trying to predict B-cell epitopes of variable lengths and applies a feature set called Composition-Transition-Distribution (CTD) which allows for the comparison of peptides of variable length by simplifying the residues into categories (38). The CTD divisions used by LBtope is based on a set proposed by Chinnasamy et al. that places each amino acid into groups (either 1, 2, or 3) based on certain physicochemical properties: hydrophobicity, polarizability, polarity, and Van der Waal’s volume (39). CTD characterizes the percent frequency of these groups (Composition), their spatial relationship to one another (Transition), and distribution of each group across the peptide (Distribution). The CTD feature set exemplifies an alternate way to encode certain physical features to allow for expanded function of an algorithm by enabling predictions on variable length peptides.

However, the difference in encoding methodologies between features can be much simpler than CTD; hydrophilicity can be calculated using a hydropathy table or a hydrophobicity index for proximal amino acids (40, 41). Hydropathy plots are frequently used to estimate hydrophobic and hydrophilic properties over a 20 amino acid window and, as such, may be an appropriate feature when an algorithm is focusing on predictions of peptides of a similar length (42, 43). Further, there is some variability between experimentally determined hydropathy tables, and pH will affect the hydrophilicity calculations of some amino acids.

Features that describe protein structure may be better at predicting conformational epitopes but may be unimportant when the algorithm is predicting a linear epitope. For example, surface accessibility and flexibility are more impactful features for B-cell epitope prediction software specializing in conformational epitopes. Sequence-based features, on the other hand, may be more beneficial in predicting linear epitopes. Physicochemical features such as hydrophobicity and polarity would be beneficial to both types of predictions and may not benefit one type of prediction more than another. In general, adding more features that provide new information about the data will improve accuracy but with certain limitations. If every feature represents a decision that can be made by a machine learning algorithm, adding noisy features increases the likelihood that a machine learning model uses a feature that appears effective in the training set but is inaccurate once extrapolated to the test set or new cases. This is because every machine learning model assigns a relative weight to different features. When a poorly correlative feature is used to make a prediction, it will likely increase the error. Typically, this is not a major concern unless the dataset is too small; too many features relative to the size of the dataset may impact the predictive accuracy of a model. Further, information provided by databases may limit the features that will be used to predict the label.

### 3.3 Algorithms – how data are processed

Another equally important aspect of a machine learning algorithm is selecting the algorithm itself. While algorithms can either be classifiers or regressors, there is much more variety to algorithms than just this single trait. We consider four common machine learning algorithms: support vector machine (SVM), neural networks, decision trees, and language models.

SVM or support vector machine is a classifier model that is accurate, simple, common, and elegant; it delineates a boundary in N-dimensional space in which data on one side of the boundary fall into one classification while data on the other side fall into a different classification. For example, if flexibility and net charge are the features for this model, every datapoint is plotted onto a grid using the flexibility score on one axis and net charge on the other. After training on the dataset, SVM algorithms will produce a line that bisects the data. If the algorithm is intended to predict B-cell epitopes, the datapoints would be classified as either “B-cell epitopes” or “non B-cell epitopes” based on this line. SVM is relatively simple computationally but is sensitive to noisy data and too many features relative to the dataset size (44, 45).

Neural networks are another common machine learning algorithm. They process data in a way that mimics how the human brain functions. Neural networks consist of a series of layers: input layer, hidden layer(s), and output layer. The input layer consists of neurons equal to the number of features and the output layer is the prediction made by the algorithm. The hidden layer(s) is defined by the programmer and is characterized by a series of weight values. The sum of all inputs multiplied by the weight values will determine whether the ‘neuron’ in the hidden layer will activate. Activated neurons will propagate data to the output layer in the same process; the hidden layer inputs are multiplied by weight values between the hidden layer and output layer. The activations in the output layer will determine the prediction of the algorithm. During training, a neural network adjusts weight values between layers to strengthen some connections and weaken others similar to how neural pathways in our brain can be reinforced or weakened. Parameters like the optimal size of the hidden layer and number of hidden layers are generally found by trail-and-error. The reason why it is difficult to predict the correct parameters is there is no way to interpret the analysis performed in these hidden layers, a black box. Two common neural networks in biological research are deep neural networks (neural networks with more than four layers) and convolutional neural networks (a neural network architecture that is effective at image processing). Convolutional neural networks can be ‘deep’. Also, neural networks rely on very large datasets and as such, trial-and-error optimization of hidden layers is very demanding computationally. While neural networks struggle with interpretability, they are highly accurate once optimized (46).

Unlike neural networks, decision trees are defined by their interpretability (47). Decision trees involve a series of nodes that each split into two ‘child’ nodes continually until they reach their pre-defined depth limit forming a pyramid shape. At each node, the algorithm imposes a criterion that splits the data into one of the two subsequent nodes. For example, if a peptide is being evaluated by the algorithm, a node could be “net charge ≥3” where all the datapoints that satisfy this criterion go to one of the child nodes and all the other datapoints go to the other child node. During training, the features at each node and the criterion are adjusted to sort the data accurately. Commonly, decision trees are used in random forests that consist of many decision trees where the output is a consensus of a majority of trees (classification), or the mean of tree outputs (regression).

Lastly, language models function by studying patterns in speech and language to predict what comes next probabilistically. These language models are also applied to analyze patterns in protein and genetic sequences to find patterns common to specific types of epitopes and produce prediction tools. Language models accurately represent the data on which they are trained but are very computationally demanding and require large datasets (48).

## 4 Applications of machine learning to epitope mapping

### 4.1 Machine learning integrated into prediction of T-cell and B-cell epitopes

Since the early 2000s, the integration of machine learning into epitope prediction has increased the accuracy of those predictions and, currently, most modern algorithms use machine learning methods (**Tables 4**, **5**). These algorithms are provided with sequence and/or structural information about a protein, and they use machine learning to predict which epitopes will be recognized by receptors on B-cells or T-cells (49).

**Table 4:** B-cell epitope prediction tools^a^.

Year	Name	Prediction specialization	Link	Machine learning	Algorithm type	Ref.
Require structural data						
2024	Discotope 3.0	N/A	https://services.healthtech.dtu.dk/services/DiscoTope-3.0/	Yes	XGBOOST	(98)
2024	SEMA 2.0 3D	Conformational	https://sema.airi.net/prediction_analysis	Yes	LM	(51)
2022	SCANNET	Conformational	http://bioinfo3d.cs.tau.ac.il/ScanNet/download.html	Yes	Deep learning	(32)
2022	Epitope3D	Conformational	https://biosig.lab.uq.edu.au/epitope3d/prediction	Yes	Adaboost Classifier	(99)
2020	EPCES	Conformational	http://sysbio.unl.edu/EPCES/	No	N/A	(24)
2019	SEPPA3.0	Conformational	http://www.badd-cao.net/seppa3/submission.html	Yes	Logistic Regression	(97)
2014	Epipred	Conformational	https://opig.stats.ox.ac.uk/webapps/sabdab-sabpred/sabpred/more#EpiPred	Yes	bespoke	(100)
2010	EPSVR	Conformational	http://sysbio.unl.edu/EPSVR/	Yes	Support Vector Regression	(24)
2008	Ellipro	Conformational	http://tools.iedb.org/ellipro/	No	N/A	(101)
Require sequence data						
2024	LBCE-BERT	Linear	https://github.com/Lfang111/LBCE-BERT	Yes	Language model	(102)
2024	CLBtope	N/A	https://webs.iiitd.edu.in/raghava/clbtope/	Yes	Random Forest	(103)
2024	SEMA 2.0 1D	Linear	https://sema.airi.net/prediction_analysis	Yes	Language model	(51)
2023	Epitope1D	Linear	https://biosig.lab.uq.edu.au/epitope1d/prediction	Yes	Explainable Boost Model	(104)
2022	BepiPred 3.0	N/A	https://services.healthtech.dtu.dk/service.php?BepiPred-3.0	Yes	Language model	(52)
2021	BCEPS	Linear	http://imath.med.ucm.es/bceps/	Yes	SVM	(105)
2021	EpiDope	Linear	https://github.com/flomock/EpiDope	Yes	DNN	(106)
2020	DLBepitope	Linear	https://bio.tools/dlbepitope	Yes	DNN	(107)
2020	AAPpred	Linear	https://www.bioinf.ru/aappred/predict	Yes	SVM	(108)
2013	Lbtope	Linear	https://webs.iiitd.edu.in/raghava/lbtope/protein.php	Yes	SVM	(27)
2012	SVMTriP	Linear	http://sysbio.unl.edu/SVMTriP/prediction.php	Yes	SVM	(109)
2010	CBTOPE	Linear	https://webs.iiitd.edu.in/raghava/cbtope/submit.php	Yes	SVM	(36)
2009	COBEPRO	Linear	https://scratch.proteomics.ics.uci.edu/	Yes	SVM	(94)
2008	BCPREDS	Linear	http://ailab-projects2.ist.psu.edu/bcpred/predict.html	Yes	SVM	(110)
2006	ABCpred	Linear	https://webs.iiitd.edu.in/raghava/abcpred/ABC_submission.html	Yes	Neural Network	(29)
2004	BCEpred	Linear	https://webs.iiitd.edu.in/raghava/bcepred/bcepred_submission.html	No	N/A	(111)
Can use either sequence or structural data						
2024	CALIBER	N/A	https://caliber.math.biu.ac.il/	Yes	Multiple ^c^	(112)
2009	EPITOPIA^b^	N/A	http://epitopia.tau.ac.il	Yes	Naïve Bayes classifier	(37)

^a^Accessibility evaluated on 12-06-2024.

^b^Epitopia is inaccessible but included because its design is discussed in this review.

^c^Users of CALIBER must select a model of Recurrent Neural Network, Graph Convolution Network, or Boosting (a combination of both).

N/A, Not applicable.

**Table 5:** T-cell epitope prediction tools^a^.

Year	Name	Link	Machine learning	Algorithm type	Ref.
MHC I predictor					
2024	TEPCAM	https://github.com/Chenjw99/TEPCAM	Yes	Deep Learning	(113)
2023	PANPEP	https://github.com/bm2-lab/PanPep	Yes	Neural Network	(114)
2023	TEINET	https://github.com/jiangdada1221/TEINet	Yes	Neural Network	(115)
2022	ATM-TCR	https://github.com/Lee-CBG/ATM-TCR	Yes	Multi-head Self-attention model	(116)
2021	TITAN	https://github.com/PaccMann/TITAN	Yes	Nearest Neighbour	(117)
2020	NETMHCpan 4.1	https://services.healthtech.dtu.dk/services/NetMHCpan-4.1/	Yes	Neural Network ^b^	(118)
2020	MHCflurry 2.0	https://github.com/openvax/mhcflurry	Yes	Logistic Regression	(119)
2019	ACME	https://github.com/HYsxe/ACME	Yes	DNN	(120)
2013	EPISOPT	http://bio.med.ucm.es/episopt.htmL	No	N/A	(121)
2009	PickPocket	https://services.healthtech.dtu.dk/services/PickPocket-1.1/	No	N/A	(122)
2007	NetCTL	https://services.healthtech.dtu.dk/services/NetCTL-1.2/	Yes	ANN	(123)
2006	EpiJen	http://www.ddg-pharmfac.net/epijen/EpiJen/EpiJen.htm	No	N/A	(124)
2005	PEPVAC	http://imed.med.ucm.es/PEPVAC/	No	N/A	(125)
MHC II predictor					
2023	MixMHX2pred	https://github.com/GfellerLab/MixMHC2pred	Yes	Neural Networks	(126)
2020	NETMHCIIpan4.0	https://services.healthtech.dtu.dk/services/NetMHCIIpan-4.0/	Yes	Neural Network ^b^	(118)
2019	MARIA	https://maria.stanford.edu/about.php	Yes	Neural Network	(127)
2019	NeonMHC2	https://neonmhc2.org/neonmhc2/neonmhc2_main/	Yes	Neural Network	(128)
2017	NNAlign 2.0	https://services.healthtech.dtu.dk/services/NNAlign-2.0/	Yes	ANN	(129)
2013	EpiDOCK	http://www.ddg-pharmfac.net/epidock/index.html	No	N/A	(130)
MHC I & MHC II predictor					
2024	TCRen	https://github.com/antigenomics/tcren-ms	No	N/A	(131)
2021	ERGO-II	https://github.com/IdoSpringer/ERGO-II	Yes	Language Model	(132)
2021	Vaxign2	https://violinet.org/vaxign/	Yes	Custom	(133)
2004	Rankpep	http://imed.med.ucm.es/Tools/rankpep.html	No	N/A	(134)
1999	SYFPEITHI	http://www.syfpeithi.de/bin/MHCServer.dll/EpitopePrediction.htm	No	N/A	(89)

^a^Accessibility evaluated on 12-06-2024.

N/A, Not applicable.

Non-machine learning methods use scoring methods to predict epitopes. In the case of EpiJen, a 4-step process produces a score and eliminates datapoints in a stepwise manner using quantitative matrices. It first determines whether proteasome cleavage would occur, then whether TAP binding would occur, then whether MHC binding would occur, and finally whether T-cell recognition would occur. The output would be a small subsection of the data.

The early B-cell epitope prediction tools focused primarily on the prediction of linear epitopes despite linear epitopes making up only a small portion of B-cell epitopes (10). Rapidly, new tools were developed that boasted higher accuracy on a benchmark dataset or pioneered new methods of analysis; for example, ABCpred was the first B-cell prediction server based on recurrent neural networks (29). Commonly, tools specialized for linear prediction take sequence files as their input and conformational predictions require structural inputs, but more recently, tools focus on predicting both linear and conformational epitopes. In 2017, BepiPred 2.0 became a benchmark algorithm for the comparison of newer ones based on its accuracy (for 2017 standards) and capability to predict both conformational and linear epitopes (50). Other tools were released since that predict both types of epitopes, including BepiPred 3.0, SEMA-1D, and SEMA-3D (51, 52). Ideally, the best tool is one that is specialized to address a given problem and trained on a dataset containing relevant cases.

The value in predicting T-cell epitopes lies in the characterization of potential immune responses prior to treatment to avoid side effects against self-peptides (53). In T-cell epitope prediction, we encounter the dichotomy of algorithms focused primarily on either MHC class I or MHC class II binding prediction (**Table 5**). The open binding groove of MHC class II molecules makes predictions much more complex compared to MHC class I. Specifically, this means that the ligand does not fit cleanly inside the binding groove; there is a structural element introduced and MHC class II predictive algorithms have lower accuracy compared to MHC class I algorithms. There exist several accessible online machine learning tools for predicting T-cell epitopes and/or MHC class I and class II peptide binding (**Table 5**). The review by Peters et al. (54) explores T-cell epitope prediction and algorithm design more thoroughly.

Since features, datasets, and algorithm selection all define the specificity of a machine learning model and the type of data it is predicting, it is difficult to meaningfully compare different T-cell and B-cell epitope prediction tools. These tools will often compare themselves to similar contemporary tools based on performance to benchmark datasets, but there are issues with generalizing the accuracy of these tools. The first issue is that the test sets used to compare algorithms to one another are not the same and often tailored to the specific comparison. For example, comparing linear B-cell epitope prediction software to conformational software would be affected by whether the benchmark dataset contained primarily linear or conformational data. If the benchmark set is small, neural networks may perform worse and if it is noisy then SVM algorithms will be disadvantaged. While this makes it difficult to easily select a single ‘best’ tool, it is important to appreciate that certain tools are highly specialized to address particular problems, and the breadth of tools available should be considered in selecting which are most appropriate.

### 4.2 Machine learning integrated into experimental epitope mapping methods

A significant advance in epitope mapping is the integration of machine learning to improve the analysis of peptide arrays (**Table 6**). For example, Xue et al. demonstrated the utility of machine learning algorithms to address a common issue for peptide arrays: certain peptides result in high noise relative to signal in the output data (low signal-to-noise ratio). This noise ratio results in difficulties for interpreting array data and affects its utility in defining the boundaries of an epitope. Often, array data is simplified to streamline interpretation; a threshold-based approach is used to convert data into a binary compatible with classification-type algorithms (20, 55–57). The data are usually sorted into either binding or not binding. However, machine learning can predict which peptides will result in a low signal to noise ratio. By training an algorithm on a small subset of the intended peptides, Xue et al. demonstrated that their machine learning program succeeded in accurately predicting which peptides in the larger peptide set would result in a low signal-to-noise ratio (58). There are several possible ways to consider predictions of which peptides will result in significant noise: ‘noisy’ peptides could be eliminated from training sets for future algorithm development, excluded from testing all together, or the predictions could be used in interpreting results.

**Table 6:** Machine learning applied to *in vitro* epitope mapping methods.

Method	Brief summary	Ref.
Peptide array	*De novo* binding prediction	(135)
	Removal of systematic effects, unreliable measurements, and non-specific secondary antibody responses	(136)
	*De novo* prediction of signal-to-noise ratios	(58)
Cryo-EM	Automated particle selection	(137, 138)
	Denoising of images	(139)
	False positive pruning in single particle analysis	(140)
	Data pre-processing	(141)
	Ice thickness determination	(142)
	Resolution estimation	(143)
	Atom structure determination	(144)
	3D model building of protein backbone	(145)
	Resolution determination improved by Alphafold 2	(146)
X-ray crystallography	Prediction of crystallizability of a protein	(147, 148)
	Crystallization outcome classification	(149)
	Model correctness	(150)
	Protein solubility prediction	(151)
	Data reduction	(152)

Machine learning is also used in docking software to predict the discrete interactions between a monoclonal antibody and a protein. Non-machine learning algorithms like Zdock and Rosetta are relatively effective and accurate antibody docking software programs, but they specialize in “local docking” which necessitates partial epitope knowledge, like relative location of the epitope within approximately 8 Å (59, 60). Machine learning allows programs like Mabtope to analyze millions of docking poses and identify those that are optimal. When compared to other methods like FRODOCK (another non-machine learning method), Mabtope reports are more accurate at predicting whether a specific residue participates in an epitope (80% accuracy compared to 35% accuracy) (61, 62). The impacts of machine learning on protein-ligand docking are discussed in a review by Yang et al. (63). Docking-based approaches provide the ability to explore specific antibody-protein interactions *in silico*.

In X-ray crystallography, machine learning can address the difficulty with performing this method on large proteins. When analyzing large proteins, a pre-experimental step can use B-cell mapping algorithms to focus on specific regions of a protein; testing a smaller discrete region of a protein would help address the size issue and improve the throughput. Machine learning can also predict protein crystallizability which saves time and resources (64, 65). Moreover, the study of highly variable regions of a protein is facilitated by algorithms that can predict ‘correctness’ scores (a per-residue estimate of how reliable the crystal structure is) for residue main chains and side chains to improve interpretability of crystallographic protein structures (**Table 6**).

Cryo-EM depends on machine learning integration where a cryogenically frozen molecule is subjected to electron microscopy to produce and assemble a 3D reconstruction. Machine learning has automated particle selection, improved post-processing resolution, and map reconstruction (**Table 6**).

## 5 Case studies

Several studies of SARS-CoV-2 highlight applications of machine learning to probe the specificity of immune responses to the virus. As an example, Hotop et al. applied machine learning to analyze data from peptide microarrays screened with sera from patients with disease outcomes of varying severity (55). The analysis revealed important differences in the antibody responses to SARS-CoV-2 that correlated with improved clearance of the infection. Unlike conventional B-cell epitope mapping algorithms that use regression analysis (providing a continuous variable as an output), this application applied a random forest in classification analysis providing an output as a ‘1’ or a ‘0’. More specifically, the machine learning algorithms labeled each peptide as either correlated (‘1’) or non-correlated (‘0’) with better disease outcomes. Five different algorithms were used to select 10 peptides (out of 648) that were recognized predominantly by sera from patients with milder infections. This information can now be applied to vaccine design to focus on neutralizing epitopes. It could also be used as a prognostic to predict the severity of disease outcomes.

A second example is the application of the algorithm ScanNet (**Table 3**) (32) to study the immune response against the receptor binding site of the SARS-CoV-2 Omicron variant (66). ScanNet is a geometric model that characterizes the binding surface of a protein (or protein segment in this case) based on its physical and chemical properties: shape, charge, depth, surface accessibility, etc. The algorithm analyzed antigenicity (the likelihood of antibody recognition) residue by residue of the SARS-CoV-2 receptor binding domain (RBD) from the original strain compared to the same region on each of the Delta, Alpha, Beta and Omicron variants. This analysis suggested that mutations in the RBD reduced antibody recognition, which could explain why immunity to previous variants of SARS-CoV-2 may not be as protective against the Omicron strain. These results were corroborated by competition ELISAs that showed reduced serological recognition of the Omicron RBD and supported the need for an Omicron-specific vaccine. ScanNet’s antigenicity predictions for the variants further supported the need to design Omicron-specific vaccines.

## 6 Future applications of machine learning to epitope mapping and immunology

Although B-cell epitope prediction algorithms are a common tool, there is room for tremendous growth in the application of machine learning to further refine epitope predictions. This is especially relevant in the interpretation of antibody-binding data that point to multiple epitopes; rather than recognizing a single discrete epitope, some antibodies are promiscuous or polyreactive (67, 68). Applying feature analysis approaches to these types of antibody binding data could identify features that are shared across epitopes within the same or related protein. An exciting opportunity is to use machine learning to extract -with high resolution - the specific features of these epitopes that are required for an antibody to bind. This information can then inform diagnostics, immunotherapeutics or vaccine design. In the example of SARS-CoV-2, peptide vaccines could be designed based on the features of an epitope that are conserved across virus variants to elicit broadly neutralizing antibodies.

Another application of machine learning is to improve prediction of conformational epitopes. High throughput mapping of linear peptide arrays could be integrated with *in silico* protein modeling, like AlphaFold, to increase the interpretability of the data (69). Recently, the release of AlphaFold 3 further improved the accuracy of predicting biomolecular complexes and overhauled its training procedure (70). Unfortunately, AlphaFold 3 is closed-source, which may limit the potential for specialized derivative models to be developed. For example, AlphaFold-Multimer was derived from the open-source code of Alphafold 2 and improved on its ability to predict large protein complexes (71). As discussed previously, peptide arrays focus on linear epitopes and are considered less informative with regards to conformational epitopes. However, mapping binding regions from an array to a predicted structure may highlight binding clusters that indicate an epitope. A more granular approach to the same methods could be to use the large peptide array datasets and train a machine learning algorithm from which feature data can be extracted. The feature data would encapsulate physical and chemical characteristics associated with binding regions (i.e. charge, secondary structure, etc.) which could also be applied to the 3D structures to identify binding sites. The impact this would have on vaccine development and therapeutics is profound. Success of these algorithms may improve peptide-based vaccines through scaffolded or stapled peptides that can recapitulate 3D structural components. It would also provide more accurate characterization of off-target binding.

A paratope-based approach may also have potential to predict epitopes and play an important role in profiling the safety and specificity of monoclonal antibodies now widely used as therapeutics. Sequences of antigen binding sites and/or structural information could be used to predict the features of a compatible epitope. In this case, the features would be structural, physicochemical, and sequence-based descriptions of the binding region of an antibody and the label(s) would correspond to aspects of an epitope, such as structure, charge, polarity, etc. Just like B-cell epitope prediction software, there are promising predictive models for paratope regions on an antibody (72–74). Additionally, programs like AlphaFold, or faster and higher-throughput derivatives, can be applied to design and inform structural features (69, 75, 76). The burgeoning field of paratope-focused analysis could yield a better understanding of off-target binding, and potentially increase accuracy and safety. Focusing on both the paratope and epitope may result in more predictable outcomes for vaccine design and other therapeutics.

Importantly, machine learning has been integrated into research beyond epitope mapping, with impacts in the broader field of immunology. For example, the tool AllerCatPro 2.0 uses machine learning to predict allergenicity of proteins by predicting potential immune epitopes (77) and, clinically, machine learning has improved allergic disease diagnostics (78). To improve resource management and streamline clinical processes, machine learning-enhanced prognostic tools are being rapidly developed to analyze patient data. Specifically, plasma from SARS-CoV-2 infected patients was used to produce a cytokine panel that can accurately predict disease severity (79). Conditions that are difficult to diagnose, such as auto immune diseases, have benefited significantly from the incorporation of machine learning models to analyze patient data (80). In future, machine learning directed to these clinical applications has the potential to transform personalized medicine.

## References

[1] Vasireddy D Vanaparthy R Mohan G Malayala SV Atluri P. Review of COVID-19 variants and COVID-19 vaccine efficacy: what the clinician should know? J Clin Med Res. (2021) 13:317–25. doi: 10.14740/jocmr4518 PMC8256910 34267839

[2] Xing K Tu XY Liu M Liang ZW Chen JN Li JJ. Efficacy and safety of COVID-19 vaccines: a systematic review. Zhongguo Dang Dai Er Ke Za Zhi. (2021) 23:221–8. doi: 10.7499/j.issn.1008-8830.2101133 PMC7969187 33691913

[3] Pollard AJ Bijker EM. A guide to vaccinology: from basic principles to new developments. Nat Rev Immunol. (2021) 21:83–100. doi: 10.1038/s41577-020-00479-7 33353987 PMC7754704

[4] Ghotloo S Maghsood F Golsaz-Shirazi F Amiri MM Moog C Shokri F. Epitope mapping of neutralising anti-SARS-CoV-2 monoclonal antibodies: Implications for immunotherapy and vaccine design. Rev Med Virol. (2022) 32:e2347. doi: 10.1002/rmv.v32.5 35394093 PMC9111153

[5] Hudson D Fernandes RA Basham M Ogg G Koohy H. Can we predict T cell specificity with digital biology and machine learning? Nat Rev Immunol. (2023) 23:511–21. doi: 10.1038/s41577-023-00835-3 PMC9908307 36755161

[6] Becker JP Riemer AB. The importance of being presented: target validation by immunopeptidomics for epitope-specific immunotherapies. Front Immunol. (2022) 13:883989. doi: 10.3389/fimmu.2022.883989 35464395 PMC9018990

[7] Sidney J Peters B Sette A. Epitope prediction and identification- adaptive T cell responses in humans. Semin Immunol. (2020) 50:101418. doi: 10.1016/j.smim.2020.101418 33131981 PMC7749839

[8] Barlow DJ Edwards MS Thornton JM. Continuous and discontinuous protein antigenic determinants. Nature. (1986) 322:747–8. doi: 10.1038/322747a0 2427953

[9] Van Regenmortel MH. What is a B-cell epitope? Methods Mol Biol. (2009) 524:3–20. doi: 10.1007/978-1-59745-450-6_1 19377933

[10] Kringelum JV Nielsen M Padkjær SB Lund O. Structural analysis of B-cell epitopes in antibody:protein complexes. Mol Immunol. (2013) 53:24–34. doi: 10.1016/j.molimm.2012.06.001 22784991 PMC3461403

[11] Rahman Kh S Chowdhury EU Sachse K Kaltenboeck B. Inadequate reference datasets biased toward short non-epitopes confound B-cell epitope prediction. J Biol Chem. (2016) 291:14585–99. doi: 10.1074/jbc.M116.729020 PMC4938180 27189949

[12] Potocnakova L Bhide M Pulzova LB. An introduction to B-cell epitope mapping and in silico epitope prediction. J Immunol Res. (2016) 2016:6760830. doi: 10.1155/2016/6760830 28127568 PMC5227168

[13] Toride King M Brooks CL. Epitope mapping of antibody-antigen interactions with X-ray crystallography. Methods Mol Biol. (2018) 1785:13–27. doi: 10.1007/978-1-4939-7841-0_2 29714009 PMC6260929

[14] Krissinel E Henrick K. Inference of macromolecular assemblies from crystalline state. J Mol Biol. (2007) 372:774–97. doi: 10.1016/j.jmb.2007.05.022 17681537

[15] Smyth MS Martin JH. x ray crystallography. Mol Pathol. (2000) 53:8–14. doi: 10.1136/mp.53.1.8 10884915 PMC1186895

[16] Wigge C Stefanovic A Radjainia M. The rapidly evolving role of cryo-EM in drug design. Drug Discovery Today Technol. (2020) 38:91–102. doi: 10.1016/j.ddtec.2020.12.003 34895645

[17] Amartely H Iosub-Amir A Friedler A. Identifying protein-protein interaction sites using peptide arrays. J Vis Exp. (2014) 93:e52097. doi: 10.3791/52097 PMC4354139 25490271

[18] Morrison KL Weiss GA. Combinatorial alanine-scanning. Curr Opin Chem Biol. (2001) 5:302–7. doi: 10.1016/S1367-5931(00)00206-4 11479122

[19] Szymczak LC Kuo HY Mrksich M. Peptide arrays: development and application. Anal Chem. (2018) 90:266–82. doi: 10.1021/acs.analchem.7b04380 PMC6526727 29135227

[20] Peng M Dou X Zhang X Yan M Xiong D Jiang R. Protective antigenic epitopes revealed by immunosignatures after three doses of inactivated SARS-CoV-2 vaccine. Front Immunol. (2022) 13:938378. doi: 10.3389/fimmu.2022.938378 36016943 PMC9397116

[21] Goodfellow I Bengio Y Courville A. Deep Learning. Cambridge, Massachusetts: The MIT Press (2016).

[22] Cukierski W. Titanic - machine learning from disaster. Delaware, USA: Kaggle (2012). Available at: https://www.kaggle.com/competitions/titanic.

[23] Kosaloglu-Yalcin Z Blazeska N Vita R Carter H Nielsen M Schoenberger S. The cancer epitope database and analysis resource (CEDAR). Nucleic Acids Res. (2023) 51:D845–D52. doi: 10.1093/nar/gkac902 PMC9825495 36250634

[24] Liang S Zheng D Yao B Zhang C. EPCES and EPSVR: prediction of B-cell antigenic epitopes on protein surfaces with conformational information. Methods Mol Biol. (2020) 2131:289–97. doi: 10.1007/978-1-0716-0389-5_16 32162262

[25] Ponomarenko JV Bourne PE. Antibody-protein interactions: benchmark datasets and prediction tools evaluation. BMC Struct Biol. (2007) 7:64. doi: 10.1186/1472-6807-7-64 17910770 PMC2174481

[26] Mintseris J Wiehe K Pierce B Anderson R Chen R Janin J. Protein-protein docking benchmark 2.0: an update. Proteins. (2005) 60:214–6. doi: 10.1002/prot.20560 15981264

[27] Singh H Ansari HR Raghava GP. Improved method for linear B-cell epitope prediction using antigen's primary sequence. PLoS One. (2013) 8:e62216. doi: 10.1371/journal.pone.0062216 23667458 PMC3646881

[28] Saha S Bhasin M Raghava GP. Bcipep: a database of B-cell epitopes. BMC Genomics. (2005) 6:79. doi: 10.1186/1471-2164-6-79 15921533 PMC1173103

[29] Saha S Raghava GP. Prediction of continuous B-cell epitopes in an antigen using recurrent neural network. Proteins. (2006) 65:40–8. doi: 10.1002/prot.21078 16894596

[30] Sarker IH. Machine learning: algorithms, real-world applications and research directions. SN Comput Sci. (2021) 2:160. doi: 10.1007/s42979-021-00592-x 33778771 PMC7983091

[31] Kulkarni-Kale U Bhosle S Kolaskar AS. CEP: a conformational epitope prediction server. Nucleic Acids Res. (2005) 33:W168–71. doi: 10.1093/nar/gki460 PMC1160221 15980448

[32] Tubiana J Schneidman-Duhovny D Wolfson HJ. ScanNet: an interpretable geometric deep learning model for structure-based protein binding site prediction. Nat Methods. (2022) 19:730–9. doi: 10.1038/s41592-022-01490-7 35637310

[33] Humphrey W Dalke A Schulten K. VMD: Visual molecular dynamics. J Mol Graph Model. (1996) 14:33–8. doi: 10.1016/0263-7855(96)00018-5 8744570

[34] Bujacz A. Structures of bovine, equine and leporine serum albumin. Acta Crystallogr D Biol Crystallogr. (2012) 68:1278–89. doi: 10.1107/S0907444912027047 22993082

[35] Rubinstein ND Mayrose I Pupko T. A machine-learning approach for predicting B-cell epitopes. Mol Immunol. (2009) 46:840–7. doi: 10.1016/j.molimm.2008.09.009 18947876

[36] Ansari HR Raghava GP. Identification of conformational B-cell Epitopes in an antigen from its primary sequence. Immunome Res. (2010) 6:6. doi: 10.1186/1745-7580-6-6 20961417 PMC2974664

[37] Rubinstein ND Mayrose I Martz E Pupko T. Epitopia: a web-server for predicting B-cell epitopes. BMC Bioinf. (2009) 10:287. doi: 10.1186/1471-2105-10-287 PMC2751785 19751513

[38] El-Manzalawy Y Dobbs D Honavar V. Predicting flexible length linear B-cell epitopes. Comput Syst Bioinf Conf. (2008) 7:121–32. doi: 10.1142/p585 PMC3400678 19642274

[39] Chinnasamy A Sung WK Mittal A. Protein structure and fold prediction using tree-augmented naive Bayesian classifier. Pac Symp Biocomput. (2004), 387–98. doi: 10.1142/9789812704856_0037 14992519

[40] Di Rienzo L Miotto M Bo L Ruocco G Raimondo D Milanetti E. Characterizing hydropathy of amino acid side chain in a protein environment by investigating the structural changes of water molecules network. Front Mol Biosci. (2021) 8:626837. doi: 10.3389/fmolb.2021.626837 33718433 PMC7954116

[41] Mant CT Kovacs JM Kim HM Pollock DD Hodges RS. Intrinsic amino acid side-chain hydrophilicity/hydrophobicity coefficients determined by reversed-phase high-performance liquid chromatography of model peptides: comparison with other hydrophilicity/hydrophobicity scales. Biopolymers. (2009) 92:573–95. doi: 10.1002/bip.21316 PMC2792893 19795449

[42] Zhao T Cheng L Zang T Hu Y. Peptide-major histocompatibility complex class I binding prediction based on deep learning with novel feature. Front Genet. (2019) 10:1191. doi: 10.3389/fgene.2019.01191 31850062 PMC6892951

[43] Eisenberg D. Three-dimensional structure of membrane and surface proteins. Annu Rev Biochem. (1984) 53:595–623. doi: 10.1146/annurev.bi.53.070184.003115 6383201

[44] Li HX Yang JL Zhang G Fan B. Probabilistic support vector machines for classification of noise affected data. Inform Sci. (2013) 221:60–71. doi: 10.1016/j.ins.2012.09.041

[45] Cervantes J Garcia-Lamont F Rodríguez-Mazahua L Lopez A. A comprehensive survey on support vector machine classification: Applications, challenges and trends. Neurocomputing. (2020) 408:189–215. doi: 10.1016/j.neucom.2019.10.118

[46] Zhang Y Tino P Leonardis A Tang K. A survey on neural network interpretability. IEEE T Em Top Comp I. (2021) 5:726–42. doi: 10.1109/TETCI.2021.3100641

[47] Costa VG Pedreira CE. Recent advances in decision trees: an updated survey. Artif Intell Rev. (2023) 56:4765–800. doi: 10.1007/s10462-022-10275-5

[48] Ofer D Brandes N Linial M. The language of proteins: NLP, machine learning & protein sequences. Comput Struct Biotec. (2021) 19:1750–8. doi: 10.1016/j.csbj.2021.03.022 PMC8050421 33897979

[49] Blythe MJ Flower DR. Benchmarking B cell epitope prediction: underperformance of existing methods. Protein Sci. (2005) 14:246–8. doi: 10.1110/ps.041059505 PMC2253337 15576553

[50] Jespersen MC Peters B Nielsen M Marcatili P. BepiPred-2.0: improving sequence-based B-cell epitope prediction using conformational epitopes. Nucleic Acids Res. (2017) 45:W24–W9. doi: 10.1093/nar/gkx346 PMC5570230 28472356

[51] Ivanisenko NV Shashkova TI Shevtsov A Sindeeva M Umerenkov D Kardymon O. SEMA 2.0: web-platform for B-cell conformational epitopes prediction using artificial intelligence. Nucleic Acids Res. (2024) 52:W533–W9. doi: 10.1093/nar/gkae386 PMC11223818 38742639

[52] Clifford JN Hoie MH Deleuran S Peters B Nielsen M Marcatili P. BepiPred-3.0: Improved B-cell epitope prediction using protein language models. Protein Sci. (2022) 31:e4497. doi: 10.1002/pro.4497 36366745 PMC9679979

[53] Koren E De Groot AS Jawa V Beck KD Boone T Rivera D. Clinical validation of the "in silico" prediction of immunogenicity of a human recombinant therapeutic protein. Clin Immunol. (2007) 124:26–32. doi: 10.1016/j.clim.2007.03.544 17490912

[54] Peters B Nielsen M Sette A. T cell epitope predictions. Annu Rev Immunol. (2020) 38:123–45. doi: 10.1146/annurev-immunol-082119-124838 PMC10878398 32045313

[55] Hotop SK Reimering S Shekhar A Asgari E Beutling U Dahlke C. Peptide microarrays coupled to machine learning reveal individual epitopes from human antibody responses with neutralizing capabilities against SARS-CoV-2. Emerg Microbes Infect. (2022) 11:1037–48. doi: 10.1080/22221751.2022.2057874 PMC9009950 35320064

[56] Hada-Neeman S Weiss-Ottolenghi Y Wagner N Avram O Ashkenazy H Maor Y. Domain-scan: combinatorial sero-diagnosis of infectious diseases using machine learning. Front Immunol. (2020) 11:619896. doi: 10.3389/fimmu.2020.619896 33643301 PMC7902724

[57] Maguy A Tardif JC Busseuil D Ribi C Li J. Autoantibody signature in cardiac arrest. Circulation. (2020) 141:1764–74. doi: 10.1161/CIRCULATIONAHA.119.044408 32312099

[58] Xue AY Szymczak LC Mrksich M Bagheri N. Machine learning on signal-to-noise ratios improves peptide array design in SAMDI mass spectrometry. Anal Chem. (2017) 89:9039–47. doi: 10.1021/acs.analchem.7b01728 PMC5588089 28719743

[59] Krawczyk K Baker T Shi J Deane CM. Antibody i-Patch prediction of the antibody binding site improves rigid local antibody-antigen docking. Protein Eng Des Sel. (2013) 26:621–9. doi: 10.1093/protein/gzt043 24006373

[60] Weitzner BD Jeliazkov JR Lyskov S Marze N Kuroda D Frick R. Modeling and docking of antibody structures with Rosetta. Nat Protoc. (2017) 12:401–16. doi: 10.1038/nprot.2016.180 PMC5739521 28125104

[61] Garzon JI Lopez-Blanco JR Pons C Kovacs J Abagyan R Fernandez-Recio J. FRODOCK: a new approach for fast rotational protein-protein docking. Bioinformatics. (2009) 25:2544–51. doi: 10.1093/bioinformatics/btp447 PMC2800348 19620099

[62] Bourquard T Musnier A Puard V Tahir S Ayoub MA Jullian Y. MAbTope: A method for improved epitope mapping. J Immunol. (2018) 201:3096–105. doi: 10.4049/jimmunol.1701722 30322966

[63] Yang C Chen EA Zhang Y. Protein-ligand docking in the machine-learning era. Molecules. (2022) 27. doi: 10.3390/molecules27144568 PMC9323102 35889440

[64] Wang HL Wang MJ Tan H Li Y Zhang ZD Song JN. PredPPCrys: accurate prediction of sequence cloning, protein production, purification and crystallization propensity from protein sequences using multi-step heterogeneous feature fusion and selection. PLoS One. (2014) 9. doi: 10.1371/journal.pone.0105902 PMC4141844 25148528

[65] Slabinski L Jaroszewski L Rychlewski L Wilson IA Lesley SA Godzik A. XtalPred: a web server for prediction of protein crystallizability. Bioinformatics. (2007) 23:3403–5. doi: 10.1093/bioinformatics/btm477 17921170

[66] Tubiana J Xiang Y Fan L Wolfson HJ Chen K Schneidman-Duhovny D. Reduced B cell antigenicity of Omicron lowers host serologic response. Cell Rep. (2022) 41:111512. doi: 10.1016/j.celrep.2022.111512 36223774 PMC9515332

[67] Jain D Salunke DM. Antibody specificity and promiscuity. Biochem J. (2019) 476:433–47. doi: 10.1042/BCJ20180670 30723137

[68] Gunti S Notkins AL. Polyreactive antibodies: function and quantification. J Infect Dis. (2015) 212 Suppl 1:S42–6. doi: 10.1093/infdis/jiu512 PMC4490204 26116731

[69] Jumper J Evans R Pritzel A Green T Figurnov M Ronneberger O. Highly accurate protein structure prediction with AlphaFold. Nature. (2021) 596:583–9. doi: 10.1038/s41586-021-03819-2 PMC8371605 34265844

[70] Abramson J Adler J Dunger J Evans R Green T Pritzel A. Accurate structure prediction of biomolecular interactions with AlphaFold 3. Nature. (2024) 630, 493–500. doi: 10.1038/s41586-024-07487-w PMC11168924 38718835

[71] Liu J Guo Z Wu T Roy RS Quadir F Chen C. Enhancing alphafold-multimer-based protein complex structure prediction with MULTICOM in CASP15. Commun Biol. (2023) 6:1140. doi: 10.1038/s42003-023-05525-3 37949999 PMC10638423

[72] Deac A VeliCkovic P Sormanni P. Attentive cross-modal paratope prediction. J Comput Biol. (2019) 26:536–45. doi: 10.1089/cmb.2018.0175 30508394

[73] Chinery L Wahome N Moal I Deane CM. Paragraph-antibody paratope prediction using graph neural networks with minimal feature vectors. Bioinformatics. (2023) 39. doi: 10.1093/bioinformatics/btac732 36370083

[74] Liberis E Velickovic P Sormanni P Vendruscolo M Lio P. Parapred: antibody paratope prediction using convolutional and recurrent neural networks. Bioinformatics. (2018) 34:2944–50. doi: 10.1093/bioinformatics/bty305 29672675

[75] Varadi M Anyango S Deshpande M Nair S Natassia C Yordanova G. AlphaFold Protein Structure Database: massively expanding the structural coverage of protein-sequence space with high-accuracy models. Nucleic Acids Res. (2022) 50:D439–D44. doi: 10.1093/nar/gkab1061 PMC8728224 34791371

[76] Mirdita M Schutze K Moriwaki Y Heo L Ovchinnikov S Steinegger M. ColabFold: making protein folding accessible to all. Nat Methods. (2022) 19:679–82. doi: 10.1038/s41592-022-01488-1 PMC9184281 35637307

[77] Nguyen MN Krutz NL Limviphuvadh V Lopata AL Gerberick GF Maurer-Stroh S. AllerCatPro 2.0: a web server for predicting protein allergenicity potential. Nucleic Acids Res. (2022) 50:W36–43. doi: 10.1093/nar/gkac446 PMC9252832 35640594

[78] Ferrante G Licari A Fasola S Marseglia GL La Grutta S. Artificial intelligence in the diagnosis of pediatric allergic diseases. Pediatr Allergy Immunol. (2021) 32:405–13. doi: 10.1111/pai.13419 33220121

[79] Patterson BK Guevara-Coto J Yogendra R Francisco EB Long E Pise A. Immune-based prediction of COVID-19 severity and chronicity decoded using machine learning. Front Immunol. (2021) 12:700782. doi: 10.3389/fimmu.2021.700782 34262570 PMC8273732

[80] Stafford IS Kellermann M Mossotto E Beattie RM MacArthur BD Ennis S. A systematic review of the applications of artificial intelligence and machine learning in autoimmune diseases. NPJ Digit Med. (2020) 3:30. doi: 10.1038/s41746-020-0229-3 32195365 PMC7062883

[81] Mendes M Mahita J Blazeska N Greenbaum J Ha B Wheeler K. IEDB-3D 2.0: Structural data analysis within the Immune Epitope Database. Protein Sci. (2023) 32:e4605. doi: 10.1002/pro.4605 36806329 PMC10022491

[82] Mamrosh E-SD-F JL Korber BTM Brander C Barouch D de Boer R Haynes BF. HIV Molecular Immunology. 2024 ed. Los Alamos, New Mexico: Los Alamos National Laboratory, Theoretical Biology and Biophysics (2023).

[83] Vita R Overton JA Greenbaum JA Ponomarenko J Clark JD Cantrell JR. The immune epitope database (IEDB) 3.0. Nucleic Acids Res. (2015) 43:D405–12. doi: 10.1093/nar/gku938 PMC4384014 25300482

[84] Olsen LR Tongchusak S Lin H Reinherz EL Brusic V Zhang GL. TANTIGEN: a comprehensive database of tumor T cell antigens. Cancer Immunol Immunother. (2017) 66:731–5. doi: 10.1007/s00262-017-1978-y PMC11028736 28280852

[85] Yang B Sayers S Xiang Z He Y. Protegen: a web-based protective antigen database and analysis system. Nucleic Acids Res. (2011) 39:D1073–8. doi: 10.1093/nar/gkq944 PMC3013795 20959289

[86] Olsen LR Zhang GL Reinherz EL Brusic V. FLAVIdB: A data mining system for knowledge discovery in flaviviruses with direct applications in immunology and vaccinology. Immunome Res. (2011) 7. PMC4276368 25544857

[87] Lata S Bhasin M Raghava GP. MHCBN 4.0: A database of MHC/TAP binding peptides and T-cell epitopes. BMC Res Notes. (2009) 2:61. doi: 10.1186/1756-0500-2-61 19379493 PMC2679046

[88] Reche PA Zhang H Glutting JP Reinherz EL. EPIMHC: a curated database of MHC-binding peptides for customized computational vaccinology. Bioinformatics. (2005) 21:2140–1. doi: 10.1093/bioinformatics/bti269 15657103

[89] Schuler MM Nastke MD Stevanovikc S. SYFPEITHI: database for searching and T-cell epitope prediction. Methods Mol Biol. (2007) 409:75–93. doi: 10.1007/978-1-60327-118-9_5 18449993

[90] Negi SS Schein CH Braun W. The updated Structural Database of Allergenic Proteins (SDAP 2.0) provides 3D models for allergens and incorporated bioinformatics tools. J Allergy Clin Immunol Glob. (2023) 2:100162. doi: 10.1016/j.jacig.2023.100162 37781674 PMC10509899

[91] Huang J Honda W. CED: a conformational epitope database. BMC Immunol. (2006) 7:7. doi: 10.1186/1471-2172-7-7 16603068 PMC1513601

[92] Schlessinger A Ofran Y Yachdav G Rost B. Epitome: database of structure-inferred antigenic epitopes. Nucleic Acids Res. (2006) 34:D777–80. doi: 10.1093/nar/gkj053 PMC1347416 16381978

[93] Blythe MJ Doytchinova IA Flower DR. JenPep: a database of quantitative functional peptide data for immunology. Bioinformatics. (2002) 18:434–9. doi: 10.1093/bioinformatics/18.3.434 11934742

[94] Sweredoski MJ Baldi P. COBEpro: a novel system for predicting continuous B-cell epitopes. Protein Eng Des Sel. (2009) 22:113–20. doi: 10.1093/protein/gzn075 PMC2644406 19074155

[95] Sun J Wu D Xu T Wang X Xu X Tao L. SEPPA: a computational server for spatial epitope prediction of protein antigens. Nucleic Acids Res. (2009) 37:W612–6. doi: 10.1093/nar/gkp417 PMC2703964 19465377

[96] Qi T Qiu T Zhang Q Tang K Fan Y Qiu J. SEPPA 2.0–more refined server to predict spatial epitope considering species of immune host and subcellular localization of protein antigen. Nucleic Acids Res. (2014) 42:W59–63. doi: 10.1093/nar/gku395 PMC4086087 24838566

[97] Zhou C Chen Z Zhang L Yan D Mao T Tang K. SEPPA 3.0-enhanced spatial epitope prediction enabling glycoprotein antigens. Nucleic Acids Res. (2019) 47:W388–W94. doi: 10.1093/nar/gkz413 PMC6602482 31114919

[98] Hoie MH Gade FS Johansen JM Wurtzen C Winther O Nielsen M. DiscoTope-3.0: improved B-cell epitope prediction using inverse folding latent representations. Front Immunol. (2024) 15:1322712. doi: 10.3389/fimmu.2024.1322712 38390326 PMC10882062

[99] da Silva BM Myung Y Ascher DB Pires DEV. epitope3D: a machine learning method for conformational B-cell epitope prediction. Brief Bioinform. (2022) 23:bbab4235. doi: 10.1093/bib/bbab423 34676398

[100] Krawczyk K Liu X Baker T Shi J Deane CM. Improving B-cell epitope prediction and its application to global antibody-antigen docking. Bioinformatics. (2014) 30:2288–94. doi: 10.1093/bioinformatics/btu190 PMC4207425 24753488

[101] Ponomarenko J Bui HH Li W Fusseder N Bourne PE Sette A. ElliPro: a new structure-based tool for the prediction of antibody epitopes. BMC Bioinf. (2008) 9:514. doi: 10.1186/1471-2105-9-514 PMC2607291 19055730

[102] Liu F Yuan C Chen H Yang F. Prediction of linear B-cell epitopes based on protein sequence features and BERT embeddings. Sci Rep. (2024) 14:2464. doi: 10.1038/s41598-024-53028-w 38291341 PMC10828400

[103] Kumar N Tripathi S Sharma N Patiyal S Devi NL Raghava GPS. A method for predicting linear and conformational B-cell epitopes in an antigen from its primary sequence. Comput Biol Med. (2024) 170:108083. doi: 10.1016/j.compbiomed.2024.108083 38295479

[104] da Silva BM Ascher DB Pires DEV. epitope1D: accurate taxonomy-aware B-cell linear epitope prediction. Brief Bioinform. (2023) 24:bbad114. doi: 10.1093/bib/bbad114 37039696 PMC10199762

[105] Ras-Carmona A Pelaez-Prestel HF Lafuente EM Reche PA. BCEPS: A web server to predict linear B cell epitopes with enhanced immunogenicity and cross-reactivity. Cells. (2021) 10. doi: 10.3390/cells10102744 PMC8534968 34685724

[106] Collatz M Mock F Barth E Holzer M Sachse K Marz M. EpiDope: a deep neural network for linear B-cell epitope prediction. Bioinformatics. (2021) 37:448–55. doi: 10.1093/bioinformatics/btaa773 32915967

[107] Liu T Shi K Li W. Deep learning methods improve linear B-cell epitope prediction. BioData Min. (2020) 13:1. doi: 10.1186/s13040-020-00211-0 32699555 PMC7371472

[108] Ya.I. Davydov AGT. Prediction of linear B-cell epitopes. Molekulyarnaya Biologiya. (2009) 43:166–74. doi: 10.1134/S0026893309010208 19334539

[109] Yao B Zhang L Liang S Zhang C. SVMTriP: a method to predict antigenic epitopes using support vector machine to integrate tri-peptide similarity and propensity. PloS One. (2012) 7:e45152. doi: 10.1371/journal.pone.0045152 22984622 PMC3440317

[110] El-Manzalawy Y Dobbs D Honavar V. Predicting linear B-cell epitopes using string kernels. J Mol Recognit. (2008) 21:243–55. doi: 10.1002/jmr.893 PMC2683948 18496882

[111] Saha S Raghava GP. (2004). ‘BCEPRED: Prediction of continuous B-cell epitopes in antigenic sequences using physico-chemical properties’, Lecture Notes in Computer Science, vol 3239. Berlin, Heidelberg: Springer. pp. 197–204. doi: 10.1007/978-3-540-30220-9_16

[112] Israeli S Louzoun Y. Single-residue linear and conformational B cell epitopes prediction using random and ESM-2 based projections. Brief Bioinform. (2024) 25:bbae084. doi: 10.1093/bib/bbae084 38487845 PMC10940830

[113] Chen J Zhao B Lin S Sun H Mao X Wang M. TEPCAM: Prediction of T-cell receptor-epitope binding specificity via interpretable deep learning. Protein Sci. (2024) 33:e4841. doi: 10.1002/pro.v33.1 37983648 PMC10731497

[114] Gao YC Gao YL Fan YX Zhu CY Wei ZT Zhou C. Pan-Peptide Meta Learning for T-cell receptor-antigen binding recognition. Nat Mach Intell. (2023) 5:236–49. doi: 10.1038/s42256-023-00619-3

[115] Jiang Y Huo M Cheng Li S. TEINet: a deep learning framework for prediction of TCR-epitope binding specificity. Brief Bioinform. (2023) 24:bbad086. doi: 10.1093/bib/bbad086 36907658

[116] Cai M Bang S Zhang P Lee H. ATM-TCR: TCR-epitope binding affinity prediction using a multi-head self-attention model. Front Immunol. (2022) 13:893247. doi: 10.3389/fimmu.2022.893247 35874725 PMC9299376

[117] Weber A Born J Rodriguez Martinez M. TITAN: T-cell receptor specificity prediction with bimodal attention networks. Bioinformatics. (2021) 37:i237–i44. doi: 10.1093/bioinformatics/btab294 PMC8275323 34252922

[118] Reynisson B Alvarez B Paul S Peters B Nielsen M. NetMHCpan-4.1 and NetMHCIIpan-4.0: improved predictions of MHC antigen presentation by concurrent motif deconvolution and integration of MS MHC eluted ligand data. Nucleic Acids Res. (2020) 48:W449–W54. doi: 10.1093/nar/gkaa379 PMC7319546 32406916

[119] O'Donnell TJ Rubinsteyn A Laserson U. MHCflurry 2.0: improved pan-allele prediction of MHC class I-presented peptides by incorporating antigen processing. Cell Syst. (2020) 11:418–9. doi: 10.1016/j.cels.2020.09.001 33091335

[120] Hu Y Wang Z Hu H Wan F Chen L Xiong Y. ACME: pan-specific peptide-MHC class I binding prediction through attention-based deep neural networks. Bioinformatics. (2019) 35:4946–54. doi: 10.1093/bioinformatics/btz427 31120490

[121] Molero-Abraham M Lafuente EM Flower DR Reche PA. Selection of conserved epitopes from hepatitis C virus for pan-populational stimulation of T-cell responses. Clin Dev Immunol. (2013) 2013:601943. doi: 10.1155/2013/601943 24348677 PMC3856138

[122] Zhang H Lund O Nielsen M. The PickPocket method for predicting binding specificities for receptors based on receptor pocket similarities: application to MHC-peptide binding. Bioinformatics. (2009) 25:1293–9. doi: 10.1093/bioinformatics/btp137 PMC2732311 19297351

[123] Stranzl T Larsen MV Lundegaard C Nielsen M. NetCTLpan: pan-specific MHC class I pathway epitope predictions. Immunogenetics. (2010) 62:357–68. doi: 10.1007/s00251-010-0441-4 PMC2875469 20379710

[124] Doytchinova IA Guan P Flower DR. EpiJen: a server for multistep T cell epitope prediction. BMC Bioinf. (2006) 7:131. doi: 10.1186/1471-2105-7-131 PMC1421443 16533401

[125] Reche PA Reinherz EL. PEPVAC: a web server for multi-epitope vaccine development based on the prediction of supertypic MHC ligands. Nucleic Acids Res. (2005) 33:W138–42. doi: 10.1093/nar/gki357 PMC1160118 15980443

[126] Racle J Guillaume P Schmidt J Michaux J Larabi A Lau K. Machine learning predictions of MHC-II specificities reveal alternative binding mode of class II epitopes. Immunity. (2023) 56:1359–75.e13. doi: 10.1016/j.immuni.2023.03.009 37023751

[127] Chen B Khodadoust MS Olsson N Wagar LE Fast E Liu CL. Predicting HLA class II antigen presentation through integrated deep learning. Nat Biotechnol. (2019) 37:1332–43. doi: 10.1038/s41587-019-0280-2 PMC7075463 31611695

[128] Abelin JG Harjanto D Malloy M Suri P Colson T Goulding SP. Defining HLA-II ligand processing and binding rules with mass spectrometry enhances cancer epitope prediction. Immunity. (2019) 51:766–79.e17. doi: 10.1016/j.immuni.2019.08.012 31495665

[129] Nielsen M Andreatta M. NNAlign: a platform to construct and evaluate artificial neural network models of receptor-ligand interactions. Nucleic Acids Res. (2017) 45:W344–W9. doi: 10.1093/nar/gkx276 PMC5570195 28407117

[130] Atanasova M Patronov A Dimitrov I Flower DR Doytchinova I. EpiDOCK: a molecular docking-based tool for MHC class II binding prediction. Protein Eng Des Sel. (2013) 26:631–4. doi: 10.1093/protein/gzt018 23661105

[131] Karnaukhov VK Shcherbinin DS Chugunov AO Chudakov DM Efremov RG Zvyagin IV. Structure-based prediction of T cell receptor recognition of unseen epitopes using TCRen. Nat Comput Sci. (2024) 4:510–21. doi: 10.1038/s43588-024-00653-0 38987378

[132] Springer I Tickotsky N Louzoun Y. Contribution of T cell receptor alpha and beta CDR3, MHC typing, V and J genes to peptide binding prediction. Front Immunol. (2021) 12:664514. doi: 10.3389/fimmu.2021.664514 33981311 PMC8107833

[133] Ong E Cooke MF Huffman A Xiang Z Wong MU Wang H. Vaxign2: the second generation of the first Web-based vaccine design program using reverse vaccinology and machine learning. Nucleic Acids Res. (2021) 49:W671–W8. doi: 10.1093/nar/gkab279 PMC8218197 34009334

[134] Reche PA Glutting JP Zhang H Reinherz EL. Enhancement to the RANKPEP resource for the prediction of peptide binding to MHC molecules using profiles. Immunogenetics. (2004) 56:405–19. doi: 10.1007/s00251-004-0709-7 15349703

[135] Taguchi AT Boyd J Diehnelt CW Legutki JB Zhao ZG Woodbury NW. Comprehensive prediction of molecular recognition in a combinatorial chemical space using machine learning. ACS Comb Sci. (2020) 22:500–8. doi: 10.1021/acscombsci.0c00003 32786325

[136] Renard BY Lower M Kuhne Y Reimer U Rothermel A Tureci O. rapmad: Robust analysis of peptide microarray data. BMC Bioinf. (2011) 12:324. doi: 10.1186/1471-2105-12-324 PMC3174949 21816082

[137] Ogura T Sato C. An automatic particle pickup method using a neural network applicable to low-contrast electron micrographs. J Struct Biol. (2001) 136:227–38. doi: 10.1006/jsbi.2002.4442 12051902

[138] Ogura T Sato C. Automatic particle pickup method using a neural network has high accuracy by applying an initial weight derived from eigenimages: a new reference free method for single-particle analysis. J Struct Biol. (2004) 145:63–75. doi: 10.1016/S1047-8477(03)00139-4 15065674

[139] Lei H Yang Y. CDAE: A cascade of denoising autoencoders for noise reduction in the clustering of single-particle cryo-EM images. Front Genet. (2020) 11:627746. doi: 10.3389/fgene.2020.627746 33552141 PMC7854571

[140] Sanchez-Garcia R Segura J Maluenda D Carazo JM Sorzano COS. Deep Consensus, a deep learning-based approach for particle pruning in cryo-electron microscopy. IUCrJ. (2018) 5:854–65. doi: 10.1107/S2052252518014392 PMC6211526 30443369

[141] Tegunov D Cramer P. Real-time cryo-electron microscopy data preprocessing with Warp. Nat Methods. (2019) 16:1146–52. doi: 10.1038/s41592-019-0580-y PMC6858868 31591575

[142] Yokoyama Y Terada T Shimizu K Nishikawa K Kozai D Shimada A. Development of a deep learning-based method to identify "good" regions of a cryo-electron microscopy grid. Biophys Rev. (2020) 12:349–54. doi: 10.1007/s12551-020-00669-6 PMC7242580 32162215

[143] Avramov TK Vyenielo D Gomez-Blanco J Adinarayanan S Vargas J Si D. Deep learning for validating and estimating resolution of cryo-electron microscopy density maps (dagger). Molecules. (2019) 24. doi: 10.3390/molecules24061181 PMC6471695 30917528

[144] Pfab J Phan NM Si D. DeepTracer for fast *de novo* cryo-EM protein structure modeling and special studies on CoV-related complexes. Proc Natl Acad Sci U.S.A. (2021) 118. doi: 10.1073/pnas.2017525118 PMC7812826 33361332

[145] Si D Moritz SA Pfab J Hou J Cao R Wang L. Deep learning to predict protein backbone structure from high-resolution cryo-EM density maps. Sci Rep. (2020) 10:4282. doi: 10.1038/s41598-020-60598-y 32152330 PMC7063051

[146] Hryc CF Baker ML. AlphaFold2 and CryoEM: Revisiting CryoEM modeling in near-atomic resolution density maps. iScience. (2022) 25:104496. doi: 10.1016/j.isci.2022.104496 35733789 PMC9207676

[147] Zhu YH Hu J Ge F Li F Song J Zhang Y. Accurate multistage prediction of protein crystallization propensity using deep-cascade forest with sequence-based features. Brief Bioinform. (2021) 22:bbaa076. doi: 10.1093/bib/bbaa076 32436937

[148] Wang PH Zhu YH Yang X Yu DJ. GCmapCrys: Integrating graph attention network with predicted contact map for multi-stage protein crystallization propensity prediction. Anal Biochem. (2023) 663:115020. doi: 10.1016/j.ab.2022.115020 36521558

[149] Bruno AE Charbonneau P Newman J Snell EH So DR Vanhoucke V. Classification of crystallization outcomes using deep convolutional neural networks. PLoS One. (2018) 13:e0198883. doi: 10.1371/journal.pone.0198883 29924841 PMC6010233

[150] Bond PS Wilson KS Cowtan KD. Predicting protein model correctness in Coot using machine learning. Acta Crystallogr D Struct Biol. (2020) 76:713–23. doi: 10.1107/S2059798320009080 PMC7397494 32744253

[151] Khurana S Rawi R Kunji K Chuang GY Bensmail H Mall R. DeepSol: a deep learning framework for sequence-based protein solubility prediction. Bioinformatics. (2018) 34:2605–13. doi: 10.1093/bioinformatics/bty166 PMC6355112 29554211

[152] Rahmani V Nawaz S Pennicard D Setty SPR Graafsma H. Data reduction for X-ray serial crystallography using machine learning. J Appl Crystallogr. (2023) 56:200–13. doi: 10.1107/S1600576722011748 PMC9901916 36777143

